# Psychological Flexibility Processes Differentially Predict Anxiety, Depression, and Well-Being Throughout Cardiac Rehabilitation

**DOI:** 10.3390/jcm14144937

**Published:** 2025-07-11

**Authors:** Chiara A. M. Spatola, Giada Rapelli, Christina L. Goodwin, Roberto Cattivelli, Giada Pietrabissa, Gabriella Martino, Gianluca Castelnuovo

**Affiliations:** 1 Department of Clinical and Experimental Medicine, University of Messina, 98125 Messina, Italy; gabriella.martino@unime.it; 2 Department of Psychology, Catholic University of the Sacred Heart, 20123 Milano, Italy; giada.rapelli@unicatt.it (G.R.); giada.pietrabissa@unicatt.it (G.P.); gianluca.castelnuovo@unicatt.it (G.C.); 3 Department of Psychiatry & Behavioral Health, Cooper University Health Care, Camden, NJ 08103, USA; goodwin-christina@cooperhealth.edu; 4 Department of Psychiatry, Cooper Medical School of Rowan University, Camden, NJ 08103, USA; 5 Department of Psychology Renzo Canestrari, Alma Mater Studiorum University of Bologna, 40127 Bologna, Italy; roberto.cattivelli@unibo.it; 6 IRCCS Istituto Auxologico Italiano, Clinical Psychology Research Laboratory, 20145 Milan, Italy

**Keywords:** psychological flexibility, cardiac rehabilitation, anxiety and depression, psychological well-being, acceptance and commitment therapy (ACT), longitudinal study

## Abstract

**Background.** Several psychological processes can influence the adjustment of cardiac patients. Psychological flexibility has been linked to significant improvements in psychological well-being during cardiac rehabilitation (CR). It can be understood as the dynamic interaction of three key processes: *openness to experience* (OE), *behavioral awareness* (BA), and *value-driven action* (VA). This study aimed to (1) evaluate the distinct role of these processes in predicting anxiety, depression, and psychological well-being in cardiac patients, and (2) assess these associations over the course of CR. **Methods.** A total of 194 CR patients participated in this longitudinal study, with 156 completing follow-up assessments at T2. Anxiety and depression were measured using the Patient Health Questionnaire-4, psychological well-being with the Psychological Well-being Index-Short, and psychological flexibility using the Comprehensive Assessment of ACT Processes. **Results.** Cross-sectional regression analysis revealed that all three psychological flexibility dimensions were negatively associated with anxiety and depression and positively associated with psychological well-being at T1. However, longitudinal analyses showed that only VA was positively associated with a decrease in depressive symptoms following CR. A sensitivity analysis conducted on the subgroup of patients with mild to severe symptoms of anxiety and depression further confirmed the robustness of these findings. **Conclusions.** These results highlight the potential benefits of measuring specific psychological flexibility processes when examining the psychological status of cardiac patients and when planning psychological interventions during CR.

## 1. Introduction

The bi-directional effect of psychological well-being on cardiovascular health is well-established [1,2] and multifactorial [2,3]. Poor mental health often coexists with unhealthy lifestyle behaviors, such as poor diet and physical inactivity, which are in turn notable risk factors for developing cardiovascular disease. As such, depression and anxiety are associated with the development [4,5,6] and maintenance [7,8] of cardiovascular disease.

Despite the literature suggesting the importance of tending to the mental health of cardiac patients, anxiety and depression remain underdiagnosed in this patient population [9], though assessment of the same is improving. The estimated prevalence of anxiety symptoms and moderate-to-severe depression symptoms at the start of CR is 28% and 18%, respectively [10], 6-times and 4-times higher (respectively) than prevalence rates found in the general population for anxiety [11] and depression [12]. CR patients with comorbid depression and anxiety are twice as likely to be non-adherent to secondary prevention efforts [13], notably increasing their risk of major adverse cardiac events and mortality [13].

Cardiac rehabilitation (CR) focuses on improving diet and increasing physical activity to improve cardiovascular outcomes; patients with poor mental health can fare worse than peers with better mental health. CR patients reporting anxiety and depression experience less functional improvement and lower exercise tolerance over the course of CR [14,15]. Improvements in mental health and well-being could lead to greater engagement in valued activities and the adoption of protective health behaviors [9]. CR could present the ideal time to foster emotional well-being in service of improved cardiovascular health and life satisfaction. The ability to accept difficult thoughts, emotions, and sensations while participating in CR may serve as a protective factor against depression and anxiety among CR patients. These skills are central to Acceptance and Commitment Therapy (ACT), which aims to increase psychological flexibility (PF).

Psychological flexibility refers to the ability to behave in a way that is consistent with one’s personal values and goals, regardless of one’s present moment internal experiences (such as thoughts, emotions, and sensations [16]. Psychological flexibility is associated with improved mental health across various medical populations [16,17,18,19,20]. A study by Ellis et al. [21] found that greater experiential avoidance and negative mood significantly predicted worse next-week cardiac rehabilitation attendance rates. Moreover, it has been shown that higher levels of psychological flexibility are longitudinally associated with greater psychological well-being among CR patients [22]. However, this study assessed experiential avoidance, a sub-process of psychological flexibility, and did not capture the totality of this complex process. Psychological flexibility could be a relevant factor in understanding the relationship between mental health and CR outcomes, however comprehensive measures of psychological flexibility have not been used with this population which limits meaningful conclusions [23].

Psychological flexibility can be conceptualized as a dynamic interaction of three dyadic processes: ‘openness to experience’ (OE); ‘behavioral Awareness’ (BA); and ‘value-driven action’ (VA) [24]. These three interrelated and mutually supportive components of psychological flexibility enable individuals to respond more effectively to life’s difficulties and discomforts—such as those experienced in the context of CR and adapting to a chronic medical condition. OE encompasses the ACT concepts of acceptance and defusion. When practicing acceptance and cognitive defusion, individuals can disengage from unhelpful thought patterns and emotional reactions, which can reduce their influence on behaviors.

BA refers to “self-awareness” and represents one’s ability to monitor their own moment-to-moment thoughts, feelings, and urges. VA refers to “motivation and activation” and represents one’s connection with one’s individual values and commitment to engaging in valued behaviors. The Comprehensive Assessment of Acceptance and Commitment Therapy Processes (CompACT; [23]) is a multidimensional measure of psychological flexibility that assesses these three dyadic processes. Rogge et al. [25] highlighted that using multidimensional measures of psychological flexibility can provide nuanced and novel results that may not be captured by adopting unidimensional psychological flexibility scales. Utilizing a multidimensional measure of psychological flexibility can provide important nuances in psychological processes that cannot be captured on unidimensional psychological flexibility scales [25]. To our knowledge, CompACT has not been used to assess psychological flexibility in the cardiac population; doing so could provide valuable insights into the coping strategies and overall well-being of CR patients.

The aims of the present study are (1) to confirm the hypothesis that psychological flexibility processes are associated with psychological health and well-being in cardiac patients; (2) to explore the degree to which each of the three dimensions of psychological flexibility uniquely predicts variance in anxiety, depression, and general psychological well-being at the beginning of CR; (3) to evaluate whether the three psychological flexibility dimensions predict improvements in anxiety, depression, and overall well-being during CR; and (4) to assess the robustness of this association within a subgroup of patients experiencing mild to severe anxiety and depression symptoms.

## 2. Methods

### 2.1. Participants

One hundred and ninety-four patients were enrolled in this longitudinal prospective study and completed baseline assessment. Participants were consecutively recruited in the context of the CR program at the Istituto Auxologico Italiano, Ospedale San Luca, located in Northern Italy.

The patients were considered eligible for the study if they met the following inclusion criteria: (1) age between 18 and 80 years; (2) affected by a cardiovascular disease; (3) fluency in spoken and written Italian language. The exclusion criteria included the presence of severe (1) psychiatric (e.g., schizophrenia), (2) cognitive (e.g., dementia), and/or (3) audio-visual (e.g., deafness) impairments. All patients provided their written consent prior to enrolment. This study was approved by the local Institutional Review Board. All procedures performed were in accordance with the 1964 Helsinki Declaration and its later amendments or comparable ethical standards.

### 2.2. The Multidisciplinary CR Program

Following the baseline assessment, participants engaged in a standardized outpatient CR program developed in alignment with EU best practice guidelines. The program lasted approximately six weeks and included structured group exercise training, one-to-one educational counselling, and periodic medical evaluations, and was conducted by a multidisciplinary team including psychologists, psychotherapists, physicians, dieticians, and physiotherapists.

Each exercise session, lasting 2 h and 15 min, began with stretching and calisthenics, followed by aerobic activities such as treadmill walking or cycling. The educational counselling, delivered by a licensed psychologist in a single 90-min one-on-one session, focused on the impact of modifiable cardiovascular risk factors and provided evidence-based behavioral strategies to effectively manage and mitigate these risks. Upon completing the program, participants underwent a post-CR assessment.

### 2.3. Assessment Measures

#### 2.3.1. Social/Demographic Data

Participants completed inventories assessing demographic information such as age, sex, marital status, education level, and employment status.

#### 2.3.2. Psychological Flexibility

Psychological flexibility was assessed using the CompACT [23]. This is a validated self-report measure of the three dyadic processes of psychological flexibility and includes 23 items scored on a seven-point Likert scale ranging from 0 (strongly disagree) to 6 (strongly agree). The CompACT-OE subscale includes ten items and scores range from 0 to 60; the CompACT-BA subscale includes five items and scores range from 0 to 30; and the CompACT-VA subscale includes eight items and scores range from 0 to 48. The CompACT has demonstrated evidence of acceptable internal consistency in a non-clinical sample of adults in the UK, with Cronbach’s alpha of 0.90 for OE, 0.87 for BA, and 0.90 for VA [23]. The validated Italian version of the COMPACT, developed after a transcultural adaptation process, has shown semantic, conceptual, and normative equivalence to the original UK scale and good content validity [26]. In the current study, the three CompACT subscales showed an acceptable to good internal consistency with a Cronbach’s alpha of 0.542 for VA, 0.850 for BA, and 0.533 for OE.

#### 2.3.3. Anxiety and Depression

Anxiety and depression symptoms were assessed using the Patient Health Questionnaire-4 (PHQ-4) [27]. The PHQ-4 is a widely accepted brief self-report measure that includes two anxiety items and two depression items. Each item is scored on a four-point Likert scale ranging from 0 (“not at all”) to 3 (“nearly every day”). The total PHQ-4 scores are categorized as normal (0–2), mild (3–5), moderate (6–8), or severe (9–12) according to a previous study [27]. In this study, we divided the sample into two groups based on the total PHQ-4 score: the “reference” group (PHQ-4 score < 3) and the “symptomatic” group (PHQ-4 score ≥ 3).

A study conducted in a large Italian sample of cardiac patients [28] showed that the PHQ-4 Italian version represents a good screening tool for measuring depression and anxiety in CVD patients. In the current study, the anxiety subscale (PHQ-Anxiety) showed a good internal consistency with a Cronbach’s alpha of 0.747, and the depression subscale (PHQ-Depression) showed an acceptable internal consistency with a Cronbach’s alpha of 0.683.

#### 2.3.4. Psychological Well-Being

The Psychological General Well-Being Index-Short (PGWBI-S) [29] was used to evaluate general psychological well-being. It consists of six components from the original survey to create a new and abbreviated summary scale. The PGWBI-S [29] has good internal consistency (0.94), comparable to the longer original measure, and demonstrated strong validity. In the current study, the scale showed a good internal consistency with a Cronbach’s alpha of 0.808. Higher scores indicate greater well-being.

### 2.4. Statistical Analyses

Descriptive statistics were used to summarize the sample characteristics. We estimated the prevalence of the risk for anxiety and depression in the total sample, in different sex and age groups.

Independent-sample *t*-tests were conducted to examine differences between completers and non-completers in psychological variables at baseline.

To evaluate the mean changes in all the study variables from pre- to post-CR, a paired-sample *t*-test was conducted in the whole sample. A sensitivity analysis was implemented to assess the mean changes only in the symptomatic subgroup of patients (i.e., patients showing mild to severe anxious or depressive symptomatology). In order to preliminarily assess the cross-sectional and longitudinal bivariate association between the three dimensions of psychological flexibility and the psychological outcomes, Pearson’s linear correlations were computed.

In order to test the predictive role of the three psychological flexibility dimensions on psychological outcomes at T1, hierarchical multiple regression analyses were run, with a two-step procedure. In the first step, age and sex were included as potential confounders (Model 1), given their well-documented associations with individual differences in anxiety, depression, and psychological well-being. In the second step, OE, BA, and VA were entered as predictors in order to test whether they explain a unique portion of the variance in addition to demographics (Model 2). In order to increase power, age was not included in the final cross-sectional regression analysis as its contribution was not significant for any of the outcome variables at T1.

Then, in order to test the prospective association of the three psychological flexibility dimensions at T1 with psychological outcomes at T2, longitudinal multiple regression analyses were run using a similar hierarchical procedure. In the first step, we controlled for the effect of age, sex, and baseline scores of the dependent outcome variable (Model 1). In the second step, the scores of OE, BA, and VA at T1 were included to test whether they significantly explain unique portions of variance (Model 2). In order to increase power, sex was not included in the final longitudinal regression analysis as its contribution was not significant for any of the outcome variables at T2.

To assess the robustness of the findings among patients with mild to severe anxious and depressive symptomatology, a sensitivity analysis was conducted by repeating the regression analyses in this specific subgroup of patients. All relevant assumptions, such as additivity, normality, linearity, homogeneity, and homoscedasticity were met. The significance threshold was set at α = 0.05. Data were analyzed using the software IBM SPSS version 22.0 (SPSS Inc., Chicago, IL, USA).

## 3. Results

A total of 342 patients were considered for eligibility, 249 patients met the inclusion criteria, and 194 agreed to participate and completed baseline assessment measures. Among these 194 CR patients, 156 also completed follow-up measurements at T2. No significant differences were found between completers and non-completers with respect to psychological variables at baseline (all *p*-values > 0.05).

### 3.1. Missing Data

There was some missing data in this two-wave study. In particular, 145 CR patients completed the COMPACT scale on psychological flexibility factors at baseline, while 128 did so at T2. The missing data may be attributed to the length of the COMPACT scale and the level of concentration required for certain items, which could be challenging for patients, particularly during a period of recovery after a cardiac event, when they may also be experiencing significant fatigue. This could impact the reliability of outcomes and the validity of conclusions drawn.

### 3.2. Descriptive Statistics

As shown in Table 1, at baseline, the study sample (*n* = 194) was mostly men (*n* = 148, 60.7%), who were engaged in an intimate relationship (62.7%). Participants’ mean age was 60.3 (*SD* = 10). Most participants had completed high school education (44%) and reported being currently employed (57%).

Based on the PHQ-4 total score, 28.2% of patients exhibited mild anxious and/or depressive symptomatology, 7.4% moderate symptomatology, and 2.1% severe symptomatology.

Table 2 shows the means and the results of paired-sample *t*-tests of outcome variables at T1 and T2. A significant change in the mean score from pre- to post-CR was observed only for psychological well-being in the full sample, with a medium effect size (Cohen’s d = −0.57). Table S1 shows the same statistics in the symptomatic subgroup of patients. Significant improvements were observed in anxiety, depression, and psychological well-being, with moderate effect sizes for anxiety (Cohen’s d = 0.36) and depression (Cohen’s d = 0.37), and a large effect size for psychological well-being (Cohen’s d = −0.81).

### 3.3. Bivariate Associations Between Study Variables

Table S2 (Supplemental Materials) reports bivariate correlations for all study variables. At baseline, there was a medium correlation between OE and BA (*r* = 0.45; *p* < 0.001) and a weak correlation between BA and VA (*r* = 0.20, *p* = 0.02), while OE and VA were not significantly correlated (*r* = 0.01, *p* = 0.897). Baseline depression and anxiety levels were significantly and largely correlated with each other (*r* = 0.65; *p* < 0.001), and both showed a significant inverse correlation with well-being (depression: *r =* −0.62, *p* < 0.001; anxiety: *r* = −0.63, *p* < 0.001). Longitudinally, significant strong correlations were observed between each variable at T1 and the same variable at T2, except for VA, which showed a weak correlation between baseline and post-CR levels (*r* = 0.30, *p* = 0.002).

Bivariate correlations between the three psychological flexibility processes and the distress outcomes at T1 and T2 are provided in Table 3. Baseline scores of OE, BA, and VA were significantly correlated with all measures of distress at both T1 and T2. The greatest effects were observed between BA at T1 and depression at T1 (*r* = −0.46, *p* < 0.001) and between VA at T1 and depression at T2 (*r* = −0.46, *p* < 0.001).

### 3.4. Cross-Sectional Multiple Regression Analyses

The results of the cross-sectional multiple regression analyses are shown in Table 4.

#### 3.4.1. Depression

After adjusting for sex and age (model 1, *R*^2^ = 0.06), the inclusion of OE, VA, and BA significantly improved the model (model 2, *R*^2^ = 0.33) and further explained 27% of the variation in depression symptoms at T1. In the final model, BA (*β* = −0.32, *p* < 0.001), VA (*β* = −0.23, *p* = 0.002), and OE (*β* = −0.20, *p* = 0.012) were all significantly associated with depression at T1.

#### 3.4.2. Anxiety

After controlling for sex and age (model 1, *R*^2^ = 0.03), OE, VA, and BA accounted for an additional 23% of the variance in anxiety symptoms at T1 (model 2, *R*^2^ =26). In the final model, VA (*β* = −0.25, *p* = 0.001), OE (*β* = −0.23, *p* = 0.005), and BA (*β* = −0.23, *p* = 0.011), showed a significant association with anxiety at T1.

#### 3.4.3. Psychological Well-Being

After adjusting for sex and age (model 1, *R*^2^ = 0.07), the inclusion of OE, VA, and BA significantly improved the model (model 2, *R*^2^ = 0.30) and further explained 23% of the variation in psychological well-being at T1. In the final model, VA (*β* = 0.28, *p* < 0.001), BA (*β* = 0.24, *p* = 0.006), OE (*β* = 0.19, *p* = 0.018), and sex (*β* = −0.16, *p* = 0.037) were significantly associated with depression at T1.

### 3.5. Longitudinal Multiple Regression Analyses

Table 5 displays the results of the longitudinal multiple regression analyses.

#### 3.5.1. Depression

After adjusting for sex, age, and depression symptoms at T1 (model 1, *R*^2^ = 0.44), the inclusion of OE, VA, and BA significantly enhanced the model (model 2, *R*^2^ = 0.49) and further explained 5% of the variation in depression symptoms at T2. In the final model, depression symptoms at T1 (*β* = 0.45, *p* ≤ 0.001), age (*β* = −0.20, *p* = 0.009), and VA (*β* = −0.22, *p* = 0.007) were significant unique predictors of T2 depression symptoms.

#### 3.5.2. Anxiety

After controlling for sex, age and anxiety symptoms at T1 (model 1, *R*^2^ = 0.48), the inclusion of OE, VA, and BA did not significantly improve the model (model 2, *R*^2^ = 0.50). In the final model (Model 1), only anxiety symptoms at T1 (*β* = 0.67, *p* < 0.001) significantly predicted anxiety at T2.

#### 3.5.3. Psychological Well-Being

After adjusting for sex, age and psychological well-being at T1 (model 1, *R*^2^ = 0.61), the inclusion of OE, VA, and BA did not significantly improve the model (model 2, *R*^2^ = 0.63). Psychological well-being at T1 (*β* = 0.74, *p* < 0.001) was the only predictor of psychological well-being at T2.

### 3.6. Sensitivity Analyses

The longitudinal regression analyses conducted on the symptomatic subgroup of patients (PHQ-4 total score ≥ 3) yielded results consistent with those observed in the whole sample across all outcome variables (see Table S3).

## 4. Discussion

In this longitudinal study of CR patients, we examined the associations between three psychological flexibility processes—openness to experience (OE), behavioral awareness (BA), and value-driven action (VA)—and anxiety, depression, and well-being over the course of CR. To our knowledge, this is the first study to explore these associations in CR patients, using a longitudinal design and a multidimensional measure of psychological flexibility. Descriptive analysis showed that 37.7% of patients exhibited mild to severe symptoms of anxiety and/or depression at the start of CR, as assessed by the PHQ-4 scale. Sensitivity analyses indicated that this symptomatic subgroup experienced significant improvements in anxiety, depression, and overall well-being from pre- to post-CR, whereas in the full sample, a significant change was observed only in psychological well-being. These findings suggest that patients with elevated psychological distress at baseline may derive greater psychological benefit from participation in CR programs. Notably, the improvements in anxiety and depression in this subgroup were associated with moderate effect sizes. Considering the relatively short duration of the CR program, it is plausible that longer interventions incorporating more intensive psychological components could yield greater improvements and larger effect sizes.

Cross-sectional regression analyses showed that all three psychological flexibility dimensions were significantly associated with psychological health at the start of CR.

To assess whether psychological flexibility dimensions can predict changes in outcome variables over the course of CR, longitudinal multiple regression analyses were performed on both the entire sample and the symptomatic patient subgroup. Baseline levels of each outcome variable (anxiety, depression, and well-being) accounted for the largest proportion of variance in their post-CR levels. However, VA contributed a significant and unique additional portion of the variance in post-CR depression, after controlling for baseline depression. In contrast, OE and BA were not significantly associated with the post-CR outcome variables. These results suggest that VA is a significant predictor of change in depression symptoms from pre- to post-CR.

This aligns with recent findings by Habibovic et al. [30], who reported that psychological flexibility components are longitudinally associated with depression but not with anxiety. In contrast, Graham et al. [31] reported that psychological flexibility prospectively predicts variance in anxiety, but not depression, in patients with muscle disorders. Our results are consistent with those of Bramwell et al. [32], who found that increases in values-based actions were significantly related to reductions in both depression and general distress among participants in an ACT program. Relatedly, a 2023 meta-analysis by Tunc et al. [33] reported a negative correlation between valued living and both depression and anxiety, with a stronger effect size for the association with depression.

VA refers to “motivation and activation” and represents one’s connection with their individual values and their commitment to engaging in valued behaviors.

Overall, our findings suggest that while all three psychological flexibility processes were cross-sectionally associated with distress outcomes before CR, only valued action uniquely contributed to the improvement of depression over time in cardiac patients. Participants who reported stronger connections to their values and values-driven behaviors at the start of CR reported lower depression scores at the program’s end. This suggests that interventions targeting valued action, such as ACT, may effectively contribute to reducing depression severity throughout CR.

A key strength of this study is the use of a multidimensional measure of psychological flexibility, which allowed us to identify specific psychological flexibility factors that uniquely impact the psychological status of CR patients. Our results demonstrated that OE, BA, and VA differentially predicted psychological outcomes, with VA emerging as the most consistent predictor. This finding supports Rogge et al. [25], who emphasized that specific dimensions of psychological flexibility differentially contribute to psychological outcomes; these are complexities that cannot be captured via unidimensional measures.

It is possible that measures of psychological flexibility used in previous studies of CR patient cardiac and mental health outcomes were not sensitive enough to detect the nuances of psychological flexibility. For instance, Spatola et al. [34] reported no significant changes in psychological flexibility levels following an ACT-based CR intervention. Although the intervention targeted all psychological flexibility components, it employed a unidimensional measure (the CVD-AAQ; [35]), which might have obscured meaningful findings. Future studies should adopt comprehensive psychological flexibility measures to assess the efficacy of ACT-based interventions and draw more nuanced conclusions.

### Strengths and Limitations of the Present Study

Several factors limit the findings of this study. First, the sample was predominantly Caucasian and male, restricting the generalizability of the results. Second, the study relied exclusively on self-reported data, which lends itself to response bias. Third, though widely utilized in research and healthcare settings, the PHQ-4 is a brief measure of anxiety and depression symptoms and does not assess full diagnostic criteria. Future research could employ various measurement approaches, such as therapist reports or indirect assessments, to strengthen the reliability of these findings.

Despite these limitations, this study highlights the importance of multidimensional psychological flexibility measurement.

Furthermore, this study is the first to explore the longitudinal association between specific ACT processes and psychological health during CR. Moreover, the sensitivity analyses suggest that the longitudinal association identified between VA and depression is robust and not driven solely by the inclusion of patients with lower levels of symptomatology. By highlighting the link between valued action and the improvement of depressive symptoms through CR, the study underscores the potential of focusing on this specific psychological flexibility process to enhance mental health outcomes in CR patients.

Increasing valued actions may help patients persist despite difficulties and setbacks during the CR program. It can give them a sense of purpose, aiding them in navigating challenges more effectively, staying committed to their therapeutic goals, and fully engaging in the therapeutic process. Valued action can therefore represent a crucial component of preventative interventions for the cardiac patients at risk of developing psychopathology.

## Figures and Tables

**Table 1:** Participant demographic characteristics at baseline (mean (SD) or *n* (%)).

Age: Mean (SD)		60.3 (10.0)
Sex: *n* (%)	Male	148 (76.3)
	Female	46 (23.7)
Education: *n* (%)	Elementary school	15 (7.8)
	Middle school	49 (25.4)
	High school	85 (44)
	College or higher	44 (22.8)
Relationship status: *n* (%)	Single	72 (37.3)
	In a relationship	121 (62.7)
Employment status: *n* (%)	Currently retired	71 (36.8)
	Currently unemployed	12 (6.2)
	Currently employed	110 (57)

**Table 2:** Means of study variables and paired-sample *t*-tests.

	*M* (*SD*) at T1	*M* (*SD*) at T2	Paired-Sample *t*-Tests (T1 vs. T2)	Cohen’s *D*
CompACT-OE	31.13 (8.72)	31.94 (9.39)	*t*_(99)_ = −1.11	−0.11
CompACT-BA	21.42 (6.68)	21.50 (7.03)	*t*_(102)_ = −0.12	−0.01
CompACT-VA	38.21 (10.36)	39.29 (10.93)	*t*_(102)_ = −0.87	−0.08
PHQ4 Depression	1.13 (1.30)	0.97 (1.18)	*t*_(149)_ = 1.78	0.14
PHQ4 Anxiety	1.29 (1.37)	1.24 (1.30)	*t*_(149)_ = 0.55	0.04
PGWBI-S	20.67 (4.96)	22.48 (4.62)	*t*_(155)_ = −7.11 **	−0.57

** *p* < 0.01; Legend: PHQ: Patient Health Questionnaire; PGWBI-S: Psychological General Well-Being Index Short form; CompACT—OE: openness to experience; CompACT—BA: behavioral awareness; CompACT—VA: value-driven action.

**Table 3:** Correlations among psychological flexibility factors at T1 and psychological outcomes at T1 and T2.

	T1			T2		
	PHQ-Depression	PHQ-Anxiety	PGWBI-S	PHQ-Depression	PHQ-Anxiety	PGWBI-S
OE at T1	−0.351 **	−0.343 **	0.304 **	−0.273 **	−0.357 **	0.339 **
BA at T1	−0.463 **	−0.372 **	0.409 **	−0.368 **	−0.301 **	0.364 **
VA at T1	−0.328 **	−0.291 **	0.358 **	−0.457 **	−0.289 **	0.383 **

** *p* < 0.01; Legend: PHQ: Patient Health Questionnaire; PGWBI-S: Psychological General Well-Being Index Short form.

**Table 4:** Cross-sectional multivariate regression analysis.

	Depression at T1					
	Model 1 *			Model 2 ^§^		
	*β*	95%CI	*p*	*β*	95%CI	*p*
Sex	0.236	[0.235, 1.244]	0.004	0.114	[−0.092, 0.802]	0.119
Age	−0.073	[−0.030, 0.011]	0.371	−0.097	[−0.031, 0.006]	0.178
CompACT-OE				−0.199	[−0.052, −0.007]	0.012
CompACT-BA				−0.323	[−0.085, −0.027]	0.000
CompACT-VA				−0.234	[−0.046, −0.011]	0.002
*R* ^2^	0.06			0.33		
*F*	4.43 **			13.69 ***		
	**Anxiety at T1**					
	**Model 1** *			**Model 2** ^§^		
	*β*	B (95%CI)	*p*	*β*	B (95%CI)	*p*
Sex	0.136	[−0.086, 0.924]	0.103	0.032	[−0.365, 0.562]	0.674
Age	−0.130	[−0.038, 0.004]	0.118	−0.137	[−0.037, 0.001]	0.071
CompACT-OE				−0.233	[−0.057, −0.010]	0.005
CompACT-BA				−0.227	[−0.069, −0.009]	0.011
CompACT-VA				−0.253	[−0.048, −0.012]	0.001
*R* ^2^	0.03			0.26		
*F*	2.41			9.94 ***		
	**Psychological well-being at T1**					
	**Model 1** *			**Model 2** ^§^		
	*β*	95%CI	*p*	*β*	B (95%CI)	*p*
Sex	−0.264	[−5.024, −1.229]	0.001	−0.156	[−3.573, −0.113]	0.037
Age	0.071	[−0.044, 0.114]	0.382	0.076	[−0.034, 0.108]	0.302
CompACT-OE				0.191	[0.018, 0.195]	0.018
CompACT-BA				0.237	[0.045, 0.269]	0.006
CompACT-VA				0.283	[0.063, 0.197]	0.000
*R* ^2^	0.07			0.30		
*F*	10.27 **			14.74 ***		

Legend: CompACT-OE: openness to experience; CompACT-BA: behavioral awareness; CompACT-VA: value-driven action.; PGWBI-S: Psychological General Well-Being Short Form; * adjusted for sex; ^§^ inclusion of PSYCHOLOGICAL FLEXIBILITY dimensions as predictors. ** = *p* < 0.01; *** = *p* < 0.000.

**Table 5:** Longitudinal multivariate regression analysis.

	Depression at T2					
	Model 1 *			Model 2 ^§^		
	*β*	B (95%CI)	*p*	*β*	B (95%CI)	*p*
PHQ Depression at T1	**0.595**	**[0.406, 0.673]**	**0.000**	**0.446**	**[0.245, 0.564]**	**0.000**
Sex	0.077	[−0.196, 0.632]	0.300	0.040	[−0.302, 0.525]	0.593
Age	**−0.203**	**[−0.40, −0.007]**	**0.006**	**−0.196**	**[−0.039, −0.006]**	**0.009**
CompACT-OE				−0.070	[−0.032, 0.012]	0.384
CompACT-BA				−0.088	[−0.042, 0.014]	0.327
CompACT-VA				**−0.219**	**[−0.040, −0.007]**	**0.007**
*R* ^2^	0.44			0.49		
*F*	28.199 ***			16,660 ***		
	**Anxiety at T2**					
	**Model 1** *			**Model 2** ^§^		
	*β*	95%CI	*p*	*β*	95%CI	*p*
PHQ Anxiety at T1	**0.669**	**[0.522, 0.802]**	**0.000**	**0.607**	**[0.437, 0.764]**	**0.000**
Sex	0.065	[−0.237, 0.642]	0.363	0.067	[−0.244, 0.663]	0.362
Age	−0.068	[−0.027, 0.009]	0.337	−0.054	[−0.025, 0.011]	0.455
CompACT-OE				−0.148	[−0.048, 0.002]	0.068
CompACT-BA				0.039	[−0.023, 0.037]	0.652
CompACT-VA				−0.071	[−0.026, 0.010]	0.361
*R* ^2^	0.48			0.50		
*F*	33.31 ***			16.34 ***		
	**Psychological well-being at T2**					
	**Model 1** *			**Model 2** ^§^		
	*β*	95%CI	*p*	*β*	95%CI	*p*
PGWBI-S at T1	**0.739**	**[0.623, 0.856]**	**0.000**	**0.725**	**[0.542, 0.822]**	**0.000**
Sex	0.007	[−1.255, 1.416]	0.905	0.007	[−1.296, 1.438]	0.918
Age	−0.025	[−0.064, 0.042]	0.682	−0.044	[−0.074, 0.035]	0.481
CompACT-OE				0.099	[−0.021, 0.127]	0.159
CompACT-BA				−0.033	[−0.110, 0.070]	0.663
CompACT-VA				0.102	[−0.014, 0.096]	0.140
*R* ^2^	0.61			0.63		
*F*	56.47 ***			28.98 ***		

Legend: CompACT-OE: openness to experience; CompACT-BA: behavioral awareness; CompACT-VA: value-driven action; PGWBI-S: Psychological General Well-Being Short Form; * adjusted for age; ^§^ inclusion of PSYCHOLOGICAL FLEXIBILITY dimensions as predictors. *** = *p* < 0.000.

## Data Availability

The dataset analyzed during the current study has been published in a public repository (and is available from the corresponding author upon reasonable request).

## Supplementary Materials

The following supporting information can be downloaded at: https://www.mdpi.com/article/10.3390/jcm14144937/s1, Table S1: Means of study variables among patients with mild to severe anxious and depressive symptoms at T1 and T2; Table S2. Bivariate correlations among all study variables. Table S3. Longitudinal multivariate regression analysis among patients with mild to severe anxious and depressive symptoms at T2.
