# Supplementary material for: Psychological Flexibility Processes Differentially Predict Anxiety, Depression, and Well-Being Throughout Cardiac Rehabilitation
Source: J Clin Med. 2025 Jul 11;14(14):4937. doi: 10.3390/jcm14144937 (PMC12296143; doi:10.3390/jcm14144937)
Supplement: Supplementary file 1 [file jcm-14-04937-s001.zip › jcm-3696350-supplementary.pdf]

**Table S1. Means of study variables among patients with mild to severe anxious and depressive symptoms at T1 and T2**

| | *M*(*SD*) at T1 | *M*(*SD*) at T2 | Paired-sample *t*-tests (T1 *vs* T2) | Cohen's *D* |
|---|---|---|---|---|
| PHQ4 Depression | 2.33(1.31) | 1.83(1.34) | **$t_{(55)}$ = 2.670; *p* = .01** | .36 |
| PHQ4 Anxiety | 2.62(1.25) | 2.18(1.49) | **$t_{(55)}$ = 2.739; *p* = .01** | .37 |
| PGWBI-S | 16.80(4.46) | 19.20(4.97) | **$t_{(55)}$ = -6.040; *p* <.001** | -.81 |

Legend: PHQ: Patient Health Questionnaire; PGWBI-S: Psychological General Well Being Index Short form.

**Table S2. Bivariate correlations among all study variables**

| | | CompACT_OE T1 | CompACT_BA T1 | CompACT_VA T1 | CompACT_OE T2 | CompACT_BA T2 | CompACT_VA T2 | PHQ4 Depression T1 | PHQ4 Anxiety T1 | PGWBI-S T1 | PHQ4 Depression T2 | PHQ4 Anxiety T2 | PGWBI-S T2 |
|---|---|---|---|---|---|---|---|---|---|---|---|---|---|
| **CompACT_OE** | *r* | 1 | .446 | .011 | .679 | .300 | .170 | -.351 | -.343 | .304 | -.273 | -.357 | .339 |
| **T1** | *p* | | .000 | .897 | .000 | .002 | .086 | .000 | .000 | .000 | .004 | .000 | .000 |
| **CompACT_BA** | *r* | .446 | 1 | .196 | .393 | .590 | .280 | -.463 | -.372 | .409 | -.368 | -.301 | .364 |
| **T1** | *p* | .000 | | .018 | .000 | .000 | .004 | .000 | .000 | .000 | .000 | .001 | .000 |
| **CompACT_VA** | *r* | .011 | .196 | 1 | .008 | .121 | .304 | -.328 | -.291 | .358 | -.457 | -.289 | .383 |
| **T1** | *p* | .897 | .018 | | .937 | .222 | .002 | .000 | .000 | .000 | .000 | .002 | .000 |
| **CompACT_OE** | *r* | .679 | .393 | .008 | 1 | .485 | .125 | -.326 | -.325 | .394 | -.314 | -.352 | .397 |
| **T2** | *p* | .000 | .000 | .937 | | .000 | .164 | .000 | .000 | .000 | .000 | .000 | .000 |
| **CompACT_BA** | *r* | .300 | .590 | .121 | .485 | 1 | .237 | -.361 | -.330 | .364 | -.292 | -.252 | .378 |
| **T2** | *p* | .002 | .000 | .222 | .000 | | .007 | .000 | .000 | .000 | .001 | .004 | .000 |
| **CompACT_VA** | *r* | .170 | .280 | .304 | .125 | .237 | 1 | -.218 | -.172 | .164 | -.237 | -.021 | .231 |
| **T2** | *p* | .086 | .004 | .002 | .164 | .007 | | .013 | .052 | .064 | .007 | .812 | .009 |
| **PHQ4 Depression** | *r* | -.351 | -.463 | -.328 | -.326 | -.361 | -.218 | 1 | .653 | -.622 | .642 | .508 | -.564 |
| **T1** | *p* | .000 | .000 | .000 | .000 | .000 | .013 | | .000 | .000 | .000 | .000 | .000 |
| **PHQ4 Anxiety** | *r* | -.343 | -.372 | -.291 | -.325 | -.330 | -.172 | .653 | 1 | -.626 | .597 | .703 | -.644 |
| **T1** | *p* | .000 | .000 | .000 | .000 | .000 | .052 | .000 | | .000 | .000 | .000 | .000 |
| **PGWBI-S** | *r* | .304 | .409 | .358 | .394 | .364 | .164 | -.622 | -.626 | 1 | -.726 | -.608 | .783 |
| **T1** | *p* | .000 | .000 | .000 | .000 | .000 | .064 | .000 | .000 | | .000 | .000 | .000 |
| **PHQ4 Depression** | *r* | -.273 | -.368 | -.457 | -.314 | -.292 | -.237 | .642 | .597 | -.726 | 1 | .661 | -.731 |
| **T2** | *p* | .004 | .000 | .000 | .000 | .001 | .007 | .000 | .000 | .000 | | .000 | .000 |
| **PHQ4 Anxiety** | *r* | -.357 | -.301 | -.289 | -.352 | -.252 | -.021 | .508 | .703 | -.608 | .661 | 1 | -.706 |
| **T2** | *p* | .000 | .001 | .002 | .000 | .004 | .812 | .000 | .000 | .000 | .000 | | .000 |
| **PGWBI-S** | *r* | .339 | .364 | .383 | .397 | .378 | .231 | -.564 | -.644 | .783 | -.731 | -.706 | 1 |
| **T2** | *p* | .000 | .000 | .000 | .000 | .000 | .009 | .000 | .000 | .000 | .000 | .000 | |

Legend. PHQ: Patient Health Questionnaire; PGWBI-S: Psychological General Well Being Index Short form; CompACT – OE: Openness to Experience; CompACT – BA: Behavioural Awareness; CompACT – VA: Valued Action.

**Table S3. Longitudinal multivariate regression analysis among patients with mild to severe anxious and depressive symptoms at T2**

| | **Depression at T2** | | | | | |
|---|---|---|---|---|---|---|
| | ***Model 1**** | | | ***Model 2*** § | | |
| | *β* | 95%CI | *p* | *β* | 95%CI | *p* |
| PHQ Depression at T1 | **.389** | **[.121, .697]** | **.007** | .223 | [-.070, .539] | .127 |
| Sex | .181 | [-.285, 1.389] | .190 | .148 | [.359, 1.262] | .266 |
| Age | **-.341** | **[ -.074, -.009]** | **.013** | **-.335** | **[-.073, -.009]** | **.013** |
| CompACT-OE | | | | -.160 | [-.095, .026] | .257 |
| CompACT-BA | | | | -.012 | [-.060, .055] | .938 |
| CompACT-VA | | | | **-.342** | **[-.093, -.011]** | **.015** |
| $R^2$ | | .36 | | | .48 | |
| *F* | | 7.12** | | | 5.44*** | |

| | **Anxiety at T2** | | | | | |
|---|---|---|---|---|---|---|
| | ***Model 1**** | | | ***Model 2*** § | | |
| | *β* | 95%CI | *p* | *β* | 95%CI | *p* |
| PHQ Anxiety at T1 | **.558** | **[.362, 1.015]** | **.000** | **.526** | **[.315, .984]** | **.000** |
| Sex | .172 | [-.298, 1.477] | .187 | .205 | [-.210, 1.619] | .127 |
| Age | -.091 | [-.048, .023] | .478 | -,033 | [-.041, .032] | .799 |
| CompACT-OE | | | | -.268 | [-.133, .003] | .061 |
| CompACT-BA | | | | .205 | [-.020, .106] | .177 |
| CompACT-VA | | | | -.088 | [-.060, .030] | .500 |
| $R^2$ | | .42 | | | .48 | |
| *F* | | 9.224*** | | | 5.502*** | |

| | **Psychological well-being at T2** | | | | | |
|---|---|---|---|---|---|---|
| | ***Model 1**** | | | ***Model 2*** § | | |
| | *β* | 95%CI | *p* | *β* | 95%CI | *p* |
| PGWBI-S at T1 | **.788** | **[.633, 1.056]** | **.000** | **.778** | **[.601, 1.067]** | **.000** |
| Sex | -.064 | [-2.918, 1.491] | .516 | -.077 | [-3.165, 1.443] | .453 |
| Age | .129 | [-.024, .140] | .159 | .104 | [-.040, .135] | .282 |
| CompACT-OE | | | | .012 | [-.157, .176] | .906 |
| CompACT-BA | | | | -.105 | [-.226, .084] | .358 |
| CompACT-VA | | | | .098 | [-.056, .166] | .323 |
| $R^2$ | | .69 | | | .71 | |
| *F* | | 44.18*** | | | 17.40*** | |

Legend: CompACT – OE: Openness to Experience; CompACT – BA: Behavioural Awareness; CompACT – VA: Valued Action.; PGWBI-S: Psychological General Well-Being Short Form; *adjusted for age; § inclusion of PF dimensions as predictors. ***$p < .001$; **$p < .01$
